# A cryopreserved and *in vivo-in vitro* validated human induced pluripotent stem cell blood-brain barrier model for reliable neurotoxicity assessment

**DOI:** 10.1016/j.namjnl.2025.100039

**Published:** 2025-07-17

**Authors:** Paul Kurtenbach, Sam Thilmany, Maria Hahn, Heidrun Ellinger-Ziegelbauer, Andreas Thomas, Marc Lamshöft, Mario Thevis

**Affiliations:** a Bayer AG Division Crop Science, Alfred-Nobel-Strasse 50, 40789 Monheim am Rhein, Germany; b Institute of Biochemistry/Center for Preventive Doping Research, German Sport University Cologne, Am Sportpark Müngersdorf 6, 50933 Cologne, Germany; c Federal Institute for Drugs and Medical Devices, Kurt-Georg-Kiesinger-Allee 3, 53175 Bonn, Germany; d Bayer AG Division Pharmaceuticals, 42096 Wuppertal, Germany; e European Monitoring Center for Emerging Doping Agents (EuMoCEDA), Cologne/Bonn, Germany

**Keywords:** Blood-brain barrier, Induced pluripotent stem cells, Cryopreservation, *In vivo-in vitro* validation, Positron emission tomography

## Abstract

Brain exposure to toxic agents is a key condition for neurotoxicity hazards in the human central nervous system (CNS). Therefore, reliable prediction of brain penetration is needed to confirm the toxicological safety of compounds to be developed. However, current regulatory risk assessments of neurotoxicity are mainly performed in rodents, which entail several critical issues associated with animal studies. Consequently, authorities worldwide have highlighted the importance of harmonized, human-relevant new approach methodologies (NAMs) such as human induced pluripotent stem cell (hiPSC)-derived models to estimate substrate permeabilities concerning the blood-brain barrier (BBB). This study aimed to redesign a commercially available human BBB model and permeability testing method for future regulatory neurotoxicity studies, considering the *in vivo* architecture and functionality as well as potential for standardization and higher throughput. Model validation should be achieved by correlation of *in vitro* permeability to human clinical positron emission tomography (PET) data to enable reliable prediction of *in vivo* brain penetration. Accordingly, cryopreserved hiPSC-derived BBB models were set up within five days in 96-transwells®, quality-controlled for *in vivo* barrier properties and exposed to seven pharmaceuticals analogously to clinical PET studies (exposure concentrations: 5 µM to 10 µM for 60 min). The results indicated that the redesigned *in vitro* permeability assessment model allows to emulate human *in vivo* brain penetration (Spearman rank correlation coefficient: 0.964). This suggests that utilizing novel commercially available, cryopreserved hiPSC-derived BBB cells could be an important starting point for a standardizable NAMs application to be implemented in regulatory neurotoxicity hazard assessment.

## Highlights

• Human blood-brain barrier (BBB) model enables prediction of *in vivo* permeability.
• High correlation of *in vitro* permeability to human clinical brain penetration data.
• Commercially available, cryopreserved BBB models can be set up within five days.
• Cryopreserved human induced pluripotent stem cells facilitate model standardization.

## Graphical abstract

A cryopreserved and *in vivo-in vitro* validated human induced pluripotent stem cell blood-brain barrier model for reliable neurotoxicity assessment. Created with Biorender.com

**Figure:**
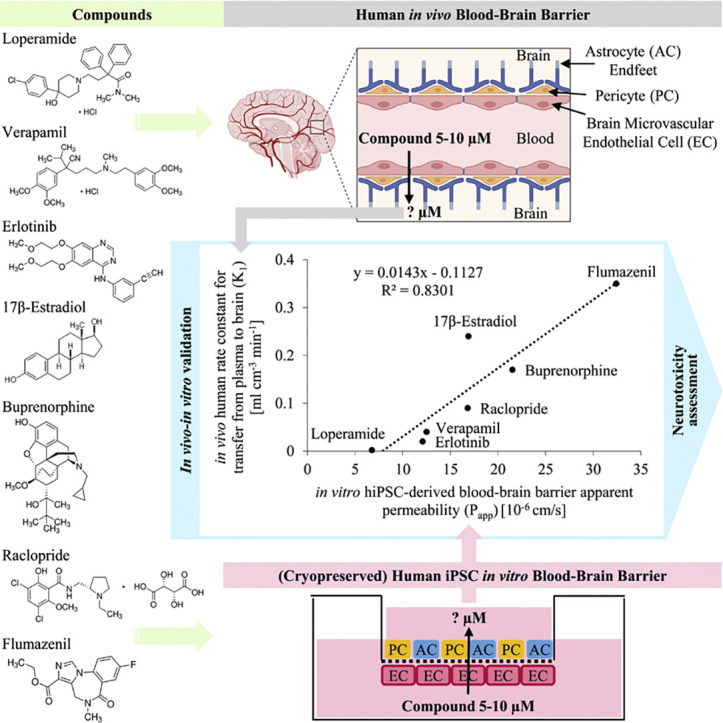

## 1 Introduction

Brain exposure to toxic compounds is a key prerequisite for neurotoxicity hazard in the human central nervous system (CNS) (Fritsche and Hogberg, 2020). Consequently, reliable prediction of brain penetration is highly desirable to assess the safety of chemicals such as pesticides (Cresto et al., 2023; Fritsche and Hogberg, 2020; Wellens et al., 2021). However, exposure prediction in current regulatory risk assessments is mainly performed in rodents, which exposes neurotoxicity testing to multiple general problems of animal testing: most importantly, pharmaceutical and chemical research has provided ample evidence showing the severe uncertainties of extrapolating animal data to humans (Bloch et al., 2024; Blum et al., 2023; Crofton et al., 2022; Fritsche and Hogberg, 2020). For instance, in the pharmaceutical industry, 19 out of 20 drug candidates fail in the clinical stage despite successful preclinical animal studies, causing $0.9 to $2.6 billion investments to be abandoned (Hartung, 2024). Notably, methods to model brain penetration appear particularly challenging as the attrition rates of CNS drugs nearly double the rate of non-CNS compounds (Talevi, 2024). For chemicals, the overwhelming time and cost of animal testing has also contributed to a wide knowledge gap represented by approximately 100 000 chemicals on the market, of which only 5 000 to 10 000 have been assessed for safety (Hartung, 2017). Within the realm of neurotoxicity, only 180 compounds worldwide have been assessed using the applicable OECD and U.S. EPA guidelines for developmental neurotoxicity (Blum et al., 2023). Finally, according to the European Commission, up to 100 000 animals per year are used only for safety assessment of pesticides, which raised large ethical issues and societal calls for change (Van der Stel et al., 2021).

As a consequence, animal testing in Europe has been banned in the cosmetics industry already since 2013, the U.S. Food and Drug Administration (FDA) has implemented a ban for the development of monoclonal antibodies in 2025, and authorities worldwide aim to replace, reduce and refine (3R) regulatory animal testing by implementation of new approach methodologies (NAMs) (Bloch et al., 2024; Escher et al., 2022; Hallier-Vanuxeem et al., 2009; U.S. Food and Drug Administration, 2025; Van der Stel et al., 2021). NAMs refer to a wide range of alternatives to animal models, such as *in vitro* models with human cells or *in silico* prediction tools based on molecular quantitative structure-activity relationships (QSAR). Regarding neurotoxicity hazard, the European Food Safety Authority (EFSA) has highlighted the importance of NAMs in modelling permeability of the blood-brain barrier (BBB), which is a key determinant of CNS exposure (Escher et al., 2022; Hallier-Vanuxeem et al., 2009).

The BBB is a complex and dynamic cellular interface between blood and CNS, which regulates nutrient supply for normal brain function as well as brain protection against neurotoxins (Sivandzade and Cucullo, 2018). It comprises multiple cell types of the neurovascular unit (NVU), including tight-junction forming brain microvascular endothelial cells (BMECs), closely associated pericytes and astrocytes (glial cells) (Abbott, 2022; Wellens et al., 2021). Apart from the physical barrier, endothelial cells lining the brain vessels possess a unique polarized expression of efflux and influx transporters (including permeability glycoprotein 1 (P-gp), multidrug resistance-associated protein 1 (MRP1) and transferrin receptor protein 1 (TFRC)), solute carriers such as glucose transporter 1 (GLUT1) as well as metabolic enzymes including cytochromes P450 (CYPs), which adds multiple functional capacities to control brain penetration of substances (Abbott, 2022; Helms et al., 2014; Stebbins et al., 2016). Consequently, a plethora of BBB *in vitro* models and corresponding validation strategies have been developed to mimic the human *in vivo* situation. Among them, only human induced pluripotent stem cell (hiPSC)-derived cells were shown to display the key physical and functional characteristics needed to model the *in vivo* BBB, while being a scalable resource as opposed to primary human cells (Abbott et al., 2022). These characteristics include transendothelial electrical resistance (TEER) > 1 000 Ω cm^2^, low paracellular permeability of fluorescent markers of 0.5 × 10^–6^ cm/s to 2 × 10^–6^ cm/s, and polarized expression of efflux transporter proteins (Abbott et al., 2022; Foreman et al., 2022). In particular, high TEER and low paracellular permeability are key prerequisites for the replication of the human *in vivo* barrier tightness (> 1000 Ω cm^2^ to 2000 Ω cm^2^) as only leak-tight models provide sufficient resolution to predict the difference between high and low permeability compounds (Abbott et al., 2022; Balzer et al., 2022).

Nevertheless, until today, no consensus model has emerged despite harmonization efforts as, for instance, promoted by the European Centre for Validation of Alternative Methods (ECVAM) (Abbott et al., 2022). The reasons include limited standardization (cell types, model architecture), long hiPSC-derived model set-up and assay times, or low throughput (Abbott et al., 2022; Escher et al., 2022). Most importantly, EFSA and academia have called for concerted efforts towards *in vitro*-*in vivo* (IVIV) validation with human-relevant data to enable reliable prediction of BBB permeability (Abbott et al., 2022; Escher et al., 2022; Fritsche and Hogberg, 2020). In this context, positron emission tomography (PET) of the human brain has been emphasized to provide a relevant but underutilized data source to assess the predictive power of human *in vitro* BBB models (Abbott et al., 2022; Le Roux et al., 2019; Mabondzo et al., 2010). PET is a noninvasive nuclear imaging technique that enables the quantification of *in vivo* brain distribution of compounds labelled with positron-emitting radionuclides (Bauer et al., 2016). If radioactivity concentrations are measured in plasma in parallel to the brain, the rate constant K_1_ for compound transfer from plasma to brain (in mL cm^-3^ min^-1^) can be calculated and used for correlation to *in vitro* permeability estimates to evaluate the predictive power of *in vitro* models (Le Roux et al., 2019; Mabondzo et al., 2010; Syvänen et al., 2022).

As a result of the above, the first objective of this study was to redesign a commercially available human *in vitro* BBB model and a permeability testing method using characterized and published cells (Jezdić et al., 2025; Stanković et al., 2025) for robust brain penetration assessment in future regulatory studies. After redesign, the model should account for the complex human BBB architecture and meet *in vivo* functional characteristics while ensuring prompt model set-up and potential for standardization and higher throughput. The second goal was to validate the cellular model with human clinical PET data to enable the reliable prediction of *in vivo* BBB permeability in regulatory neurotoxicity hazard assessment.

## 2 Materials and methods

### 2.1 Human iPSC-derived BBB model cultivation process

All human iPSC-derived blood-brain barrier cells (Brain Microvascular Endothelial Cells (BMECs), Astrocytes and Pericytes (AP)), media (BMEC maintenance medium and AP medium), and media supplements (plating supplement 500X) have been purchased from Fujifilm Cellular Dynamics Inc. (FCDI, Madison, WI, US, catalogue: R1241). The respective certificates of analysis, including cell characterization data, can be accessed in Supplementary Data File 1. All cells were derived from the same proprietary male donor. Initial TEER and paracellular permeability assays were performed using 24-transwell® PET membranes with 0.4 µm pore size (Corning, Berlin, Germany, 3472) to compare the iPSC and cell line model as well as 96-transwell® PET membranes with 1.0 µm pore size (Corning, 7369). All additional experiments included 96-transwell® membranes only. BMEC Collagen IV-Fibronectin coating solution contained 4 parts 1 mg/mL human Collagen IV (Sigma-Aldrich, Taufkirchen, Germany, C5533) in DPBS (Thermo Fisher Scientific, Schwerte, Germany 14,190,144), 1 part 1 mg/mL human Fibronectin (Sigma-Aldrich, F2006) in DPBS, and 5 parts DPBS. AP coating was performed using 0.1 % Gelatin solution in H_2_O (STEMCELL Technologies, Cologne, Germany, 07,903). Final plating media contained 1X plating supplement with 1 % Penicillin-Streptomycin (P/S, Thermo Fisher Scientific, 15,140) for BMECs and 0.5 % P/S for APs. BMEC maintenance medium contained only 1 % P/S without supplements. Unless stated differently, transwell® compartment volumes were used as recommended by Corning including 75 µL apically and 235 µL basolaterally for 96-transwells® as well as 300 µL apically and 1000 µL basolaterally for 24-transwells®.

**Fig. 1:**
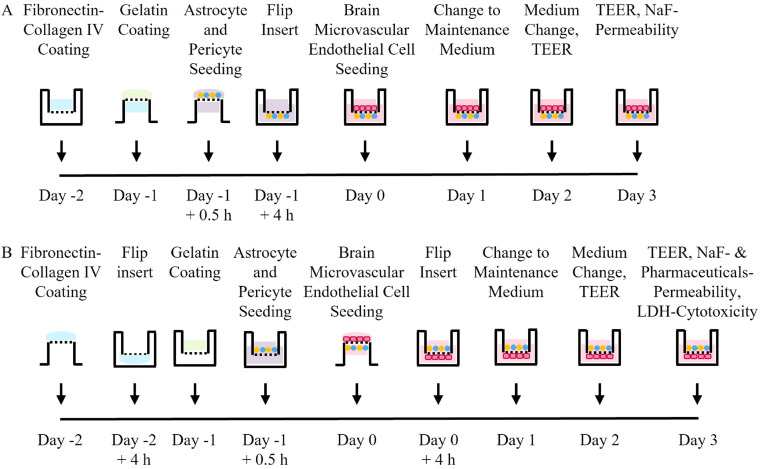
Cultivation process of tricellular hiPSC-derived blood-brain barrier (BBB) models for brain penetration assessment. (A) 24-transwell® model with brain microvascular endothelial cells (BMECs) in the apical (donor) compartment mimicking the blood side of the BBB and astrocytes and pericytes (APs) in the basolateral (acceptor) compartment emulating the brain side. (B) 96-transwell® model for higher throughput and application in micro physiological systems (MPS) with APs in the apical (acceptor) compartment and BMECs on the basolateral (donor) compartment.

On day −2, 24-transwells® were coated apically with Collagen IV-Fibronectin coating solution and stored overnight at 4 °C (Fig. 1A). In contrast, 96-transwells® were first flipped upside down, and coated with 50 µL Fibronectin-Collagen IV solution on the basolateral side for 4 h at 37 °C and 5 % CO_2_ and stored overnight at 4 °C (Fig. 1B). On day −1, 24-transwells® were flipped upside down and coated with 100 µL of a 0.1 % Gelatin solution for 0.5 h at room temperature (RT) before replacement with 100 µL of the AP seeding solution containing 1.1 × 10^6^ astrocytes/mL and 2.2 × 10^6^ pericytes/mL in AP plating medium. After seeding, cells were incubated to attach for 4 h at 37 °C and 5 % CO_2_ and then flipped back to normal orientation. On the same day, 96-transwells® were first flipped to normal orientation before being subject to 0.1 % Gelatin coating and AP seeding (6.36 × 10^5^ astrocytes/mL and 1.27 × 10^6^ pericytes/mL). In both cases, Collagen IV-Fibronectin coating solution was replaced with AP plating medium. On day 0, after 24 h at 37 °C and 5 % CO_2_, BMECs were seeded at 7.15 × 10^5^ BMECs/mL in BMEC plating medium into the apical compartment of 24-transwells®. Analogously, 96-transwells® were flipped upside down to seed endothelial cells at 1.859 × 10^6^ BMECs/mL in 50 µL BMEC plating medium onto the basolateral side. Here, cells were incubated to attach for 4 h at 37 °C and 5 % CO_2_ before flipping the transwells® slowly back to normal orientation. To inspect the cell layers for obvious leakages or cell detachment, blank transwell® filters and fully assembled models were observed under a Zeiss Primovert microscope (Carl Zeiss Microscopy GmbH, Jena, Germany) (Supplementary Figure 1). On day 1, BMEC plating medium was replaced by BMEC maintenance medium. After 24 h at 37 °C and 5 % CO_2_ barrier integrity was evaluated to consider only highly restrictive BBB models for pharmaceutical permeability testing.

### 2.2 Immortalized cell line BBB model setup

The immortalized human cerebral microvascular endothelial cell line (hCMEC/D3) was acquired from Sigma-Aldrich (SCC066). Cells were cultured in EGM-2 MV Microvascular Endothelial Growth Medium-BulletKit (Lonza, Cologne, Germany, CC-3202) supplemented with 0.1 % human Epidermal Growth Factor (hEGF), 0.1 % Vascular Endothelial Growth Factor (VEGF), 0.1 % R3-Insulin-like Growth Factor-1 (R3-IGF-1), 0.1 % Ascorbic Acid, 0.04 % Hydrocortisone, 0.4 % human Fibroblast Growth Factor Beta (hFGF-β), 5 % Fetal Bovine Serum (FBS) and 0.1 % Gentamicin/Amphotericin-B (GA). All assays were performed using 24-transwell® PET membranes with 0.4 µm pore size (Corning, 3470). Collagen coating solution contained 50 µg/mL Collagen I from calf skin (Sigma-Aldrich, C8919) in DPBS. Fibronectin coating solution contained 10 µg/mL bovine Fibronectin (Sigma-Aldrich, F1141) in DPBS. Unless stated otherwise, transwell® compartment volumes were 300 µL apically and 1 000 µL basolaterally.

The hCMEC/D3-BBB model was set up as previously described (Mursaleen et al., 2021). Briefly, the apical 24-transwells® were coated with Collagen I (1 h) and Fibronectin (1 h) at RT. As soon as cell cultures reached at least 70 % confluence in T75 flasks, cells were detached using trypsin and seeded into the apical compartment at 3.3 × 10^5^ cells/mL. After 24 h at 37 °C and 5 % CO_2_ the cell line BBB model was subject to the same tight barrier integrity testing as hiPSC-derived BBB models.

### 2.3 Characterization of barrier integrity

To assess the restrictive capacity of *in vitro* BBB models, barrier integrity was investigated in terms of TEER and paracellular marker permeability. An initial monitoring of TEER was performed from day 2 until day 7 after seeding in triplicate 24-transwells® and quadruplicate 96-transwells®, utilizing an EVOM 3 Volt/Ohm meter by World Precision Instruments (WPI, Friedberg, Germany) with a STX HTS electrode for 96-transwells® and a STX2-Plus electrode (WPI) for 24-tranwells. Maintenance and calibration were performed as recommended by the supplier. TEER was calculated as the product of electrical resistance and the respective surface area. BBB-models displaying an *in vivo*-like TEER after subtraction of blank transwells® of at least 1 000 Ω cm^2^ (Abbott et al., 2022) were considered sufficiently restrictive for permeability testing and subsequent *in vivo-in vitro* validation.

Paracellular marker permeability was investigated as previously described for hiPSC-derived models with sodium fluorescein in 24- (Stebbins et al., 2016) and 96-transwell® format (Koenig et al., 2022) with minor adaptations. In both cases, 10 µM fluorescein (Sigma-Aldrich, F6377, CAS No. 518–47–8) in BMEC Maintenance Medium were applied in the donor compartment containing BMECs. After 60 min at 37 °C, 5 % CO_2_ and 100 rpm shaking on an orbital shaker, 50 µL were sampled from the donor and acceptor (containing APs) compartment. Following a transfer to 96-well plates, samples were diluted 1:1 with 50 µL of DPBS. Fluorescence was measured with a SPARK microplate reader (TECAN, Männedorf, Switzerland) with 485/20 nm excitation and 530/20 nm emission. The apparent permeability coefficient P_app_, expressed in cm/s, was calculated as previously published (Le Roux et al., 2019):

(1) $$P_{\text{app}}\;=\left(\frac{c_{\text{ac}}}{t\cdot A}\right)\cdot \left(\frac{V_{\text{ac}}}{c_{\text{dc}}\;}\right)$$

With $c_{\text{ac}}$ being the analyte concentration in the acceptor compartment (µM), t the duration of the incubation (s, here 3 600 s), A the surface area of the respective transwell® membrane (cm^2^), $V_{\text{ac}}$ the volume of the acceptor compartment (mL) and $c_{\text{dc}}$ the applied concentration in the donor compartment (µM). $P_{\text{app}}$ was reported as the average of triplicate 24-transwell® models and six 96-transwell® model replicates. Barrier integrity was evaluated as sufficiently high when $P_{\text{app}}$ was below 2 × 10^–6^ cm/s (Abbott et al., 2022).

For the cell line model, paracellular permeability was examined using lucifer yellow (Sigma-Aldrich, L0259, CAS No. 67,769–47–5) as the typical paracellular marker for hCMEC/D3 BBB models (Mursaleen, 2021). The assay was performed as described for the hiPSC-derived model, with the only exception that fluorescence was read with 485/20 nm excitation and 527/20 nm emission. $P_{\text{app}}$ was calculated as the average of quadruplicate models.

### 2.4 Permeability testing for *in vivo*-*in vitro* validation

After evaluation of barrier integrity only the 96-transwell® hiPSC-derived model was considered to provide sufficient throughput of replicates and *in vivo*-like barrier properties to be suitable for permeability assessment and validation with human *in vivo* data. Accordingly, permeability assays were performed as follows: if models passed TEER and paracellular permeability benchmarks on day 3, the experiment was initiated by replacement of medium in the donor compartment with fresh medium spiked with 5 µM Loperamide (Sigma-Aldrich, L0750000, CAS No. 34,552–83–5, purity > 98 %), 5 µM Verapamil (Sigma-Aldrich, V4629, CAS No. 152–11–4, purity > 99 %), 5 µM Erlotinib (Sigma-Aldrich, SML3621, CAS No. 183,321–74–6, purity > 98 %), 5 µM Raclopride (Sigma-Aldrich, R121, CAS No. 98,185–20–7, purity > 97 %), 5 µM Flumazenil (Sigma-Aldrich, 5.05991, CAS No. 78,755–81–4, purity > 99 %), 10 µM 17β-Estradiol (Sigma-Aldrich, 3301, CAS No. 50–28–2, purity > 97 %) or 5 µM Buprenorphine (LGC, Wesel, Germany, LGCFOR0120.00, CAS No. 52,485–79–7, purity > 99 %). As a use-case, the permeability of the radioactively labelled pesticide [benzyl-^14^C]-Deltamethrin (4417 Bq/mL (3 µM) in medium, specific activity: 4.18 MBq/mg, CAS No. 52,918–63–5, purity > 98 %) was investigated. Additional compound data, including final solvent concentrations and physicochemical properties, were summarized in Supplementary Table 1. After 60 min at 37 °C, 5 % CO_2_ and 100 rpm shaking on an orbital shaker, 50 µL were sampled from the acceptor and donor compartments of quadruplicate BBB models as well as cell-free controls. The incubation time (exposure duration) and exposure concentration were selected analogously to clinical human PET studies used for *in vivo-in vitro* validation with mean plasma concentrations between 5 µM and 10 µM and 60 min PET scans (Bauer et al., 2015; Bauer et al., 2016).

#### 2.4.1 Sample preparation

All samples except for Deltamethrin were measured by liquid chromatography high- resolution mass spectrometry (LC—HRMS/MS). Due to handling restrictions of narcotics, the experiments including Buprenorphine (and 17β-Estradiol) were performed and analytically processed at the Institute of Biochemistry of the German Sport University Cologne. Here, 5 µL Buprenorphine-D_4_ (Sigma, B-901, CAS No. 136,781–89–0, purity > 97 %) and Estrone-D_4_ (LGC, DRE-C13213235, CAS No. 52,866–34–5, purity > 97 %) were added to Buprenorphine and 17β-Estradiol samples as internal standard (final concentration: 100 ng/mL). Then, all samples were subject to protein precipitation with acetonitrile:methanol 50:50 (v/v). After centrifugation for 10 min at 10 000 × *g*, supernatants were transferred to new vials and subsequently evaporated to dryness. Dry Buprenorphine samples were reconstituted in an acetonitrile-H_2_O (20:80, v/v) solution before analysis by LC—HRMS/MS. To derivatize 17β-Estradiol, dry samples were reconstituted with 20 µL of 1 mg/mL 1,2-Dimethyl-1H-imidazole-5-sulphonyl chloride (DMIS, LGC, TRC-D487580, CAS No. 849,351–92–4, purity > 95 %) suspension in acetone as well as 30 µL of 50 mM sodium hydrogen carbonate (Sigma-Aldrich, 1.06329, CAS No. 144–55–8, purity > 99 %) in H_2_O. Derivatization was executed in a ThermoMixer C (Eppendorf, Hamburg, Germany) at 65 °C and 600 rpm for 15 min. After evaporating samples to dryness again, final reconstitution was performed as described above. For all analytes, a volume of 5 µL was injected into the LC—HRMS/MS system. Samples containing Loperamide, Verapamil, Erlotinib, Raclopride, and Flumazenil were measured without extraction procedures.

The radioactivity analysis of [benzyl-^14^C]-Deltamethrin was carried out by liquid scintillation counting (LSC). Before the analysis, 22 µL of [benzyl-^14^C]-Deltamethrin sample volume was added to 2 mL Rotiscint High Capacity (Carl Roth, Karlsruhe, Germany, 1P1C.2).

#### 2.4.2 Sample analysis

The LC—HRMS/MS analysis was performed on an Agilent 1290 Infinity LC system (Agilent Technologies, Waldbronn, Germany) coupled to a Q Exactive Plus – Orbitrap mass spectrometer (Thermo Fisher Scientific, Bremen, Germany). Chromatographic separation was performed by means of an EC 150/2 Nucleoshell RP 18 column (2.7 μm, 2.0 × 150 mm) (Macherey-Nagel, Düren, Germany, 763,136.20) with a flow rate of 0.3 mL/min and the following 13 min-gradient comprising solvent A (H_2_O with 0.1 % formic acid) and B (acetonitrile with 0.1 % formic acid): 0.5 min 5 % solvent B, gradual increase over 9.5 min to 95 % B, steady 95 % B for 3 min and finally 1 min with 5 % B. In the MS, ionization was achieved using a heated electrospray ion (HESI) source in positive and negative (2.7 kV) ion mode. The ion transfer tube and vaporizer temperature were kept at 325 °C. The precursor ion isolation window was set to 1 *m/z*. Overall, two experiments were performed, including one full scan with an orbitrap resolution of 35 000 full width at half maximum (FWHM) at *m/z* 200 over a scan range of 50 *m/z* to 750 *m/z*, and one MS^2^ experiment in positive ionization mode with resolution 35 000 FWHM at *m/z* 200. The key LC—HRMS parameters for all analytes are summarized in Table 1. Buprenorphine and 17β-Estradiol were analyzed using a Vanquish LC system coupled to an Orbitrap Exploris 480 mass spectrometer (Thermo Fisher Scientific, Bremen, Germany). Chromatographic separation was performed on an InfinityLab Poroshell 120 EC—C18 column (2.7 μm, 3.0 × 50 mm) (Agilent Technologies, Santa Clara, *CA*, USA, 699,975–302T) with a flow rate of 0.4 mL/min and the following 11 min-gradient of solvent A (H_2_O with 0.1 % formic acid) and B (acetonitrile with 0.1 % formic acid): 0.5 min 20 % solvent B, gradual increase over 8 min to 100 % B, steady 100 % B for 1.5 min and finally 1 min with 20 % B. In the MS, ionization was achieved using a HESI source in positive (3.5 kV) and negative (-3.0 kV) ion mode. The ion transfer tube and vaporizer temperature were kept at 300 °C. The precursor ion isolation window was set to 1 *m*/*z*. Overall, three experiments were performed, including one full scan with an Orbitrap resolution of 60 000 FWHM over a scan range of 100 *m*/*z* to 500 *m*/*z*, and two MS^2^ experiments in positive ionization mode with a resolution of 15 000 FWHM. Radioactive analysis of [benzyl-^14^C]-Deltamethrin was executed using a TriCarb 2910 TR liquid scintillation analyzer (PerkinElmer, Rodgau, Germany) for 20 min and a quench curve designed for biological matrices (quench indicator tSIE/AEC: 534.30, 497.73, 295.74, 207.62, 156.16, 128.32, 107.42, 92.89, 82.55, 59.54). To achieve low counting uncertainty, the 2-sigma percent threshold was set to an error of 0.5 %. When the measurement time limit was reached before the targeted error, the individual 2-sigma percent error was reported for the respective measurement.

**Table 1:** Summary of LC-MS parameters and analyte characteristics per analyte and internal standard.

Analyte	Precursor Ion [*M* + H]^+^ [*m/z*]	Quantifier Ion [*m/z*]	Qualifier Ion [*m/z*]	Retention Time (RT) [min]	Normalized Collision Energy (NCE) [eV]
Loperamide C_29_H_33_ClN_2_O_2_	477.2303	477.2303	/	6.10	26
Verapamil C_27_H_38_N_2_O_4_	455.2904	455.2904	/	5.51	35
Erlotinib C_22_H_23_N_3_O_4_	394.1761	394.1761	/	5.00	42
Raclopride C_15_H_20_Cl_2_N_2_O_3_	347.0924	347.0924	/	4.95	40
17β-Estradiol (DMIS) C_23_H_30_N_2_O_4_S	431.1999	367.2380	96.0682	5.61	40
Buprenorphine C_29_H_41_NO_4_	468.3108	396.2169	414.2639	3.8	60
Flumazenil C_15_H_14_FN_3_O_3_	304.1092	304.1092	/	5.15	30
Estrone-D_4_ (DMIS) C_23_H_24_D_4_N_2_O_4_S	433.2094	369.2475	96.0682	5.96	40
Buprenorphine-D_4_ C_29_H_37_D_4_NO_4_	472.3359	400.2420	414.2639	3.82	60

#### 2.4.3 Quantification and correlation to clinical data

All compounds except Deltamethrin were quantified utilizing a standard calibration series, and quality control samples prepared in a blank matrix. For each substance, an individual concentration range was established. Method characterization, including linearity, accuracy, and precision, was performed based on the analytical requirements issued by the German Society of Toxicological and Forensic Chemistry (GTFCh) (Supplementary Data File 2) (Peters et al., 2009). Raw data was processed for quantification using the Quan Browser in Xcalibur (version 2.2) by Thermo Fisher Scientific. Peak areas were plotted against nominal concentrations to set up standard calibration curves, and linearity was confirmed by a coefficient of determination (R^2^) above 0.99. The respective linear functions were then used to calculate the analyte concentrations of unknown samples. For Buprenorphine and 17β-Estradiol, the peak area ratios of analyte and internal standard were used analogously. The quantification of deltamethrin samples was based on the ratio of measured radioactivity in individual samples to the application stock.

After quantification of compound concentrations, *in vitro* $P_{\text{app}}$ was calculated for each compound according to the permeability formula described in Section 2.3. To evaluate the capacity of the *in vitro*-BBB model to predict the *in vivo* permeability of substances, the *in vitro* $P_{\text{app}}$ was plotted against published clinical human *in vivo* $K_{1}$ (compound-specific rate constant for the transfer from blood across the BBB into the brain) data derived from PET compartmental modeling (Ghazanfari et al., 2024; Le Roux et al., 2019). Consequently, the Spearman rank correlation coefficient, as well as the R^2^ of the resulting linear function, were calculated. The linear model was used to predict the *in vivo* permeability of [benzyl-^14^C]-Deltamethrin.

### 2.5 LDH-cytotoxicity

To ensure that the compounds used in this study would not impact permeability through cytotoxic effects on the BBB-cells, a lactate dehydrogenase (LDH)-cytotoxicity assay (Thermo Fisher Scientific, C20301) was performed immediately after the permeability studies. The assay was executed according to the manufacturer’s instructions with adaptations to the transwell® setting for two representative high-permeability compounds (Buprenorphine and 17β-Estradiol): maximum LDH release was achieved by replacing 50 % of apical and basolateral medium volumes with lysis buffer. After incubation for 45 min at 37 °C, 5 % CO_2_, 50 µL were sampled from both compartments of all samples. Absorbance was measured at 450 and 690 nm on a microplate reader. All sample readings were corrected for background signal (690 nm reading) and blank measurements. Cytotoxicity of all samples was calculated as the percentage of maximum LDH release in apical and basolateral compartments.

## 3 Results

### 3.1 Characterization of *in vitro* BBB models

**Fig. 2:**
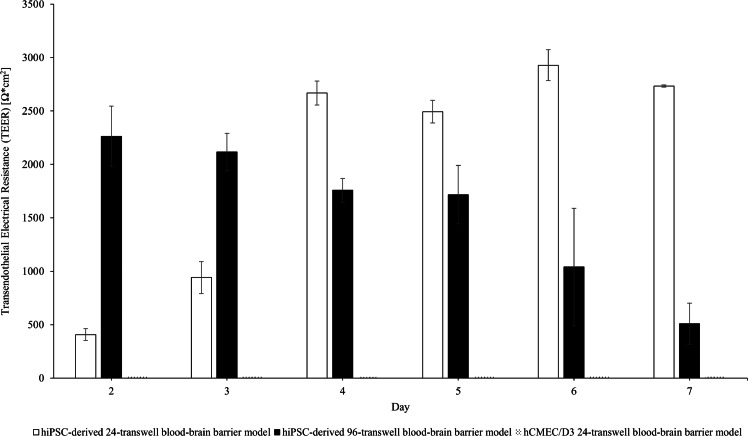
Transendothelial electrical resistance (TEER) of hiPSC-derived and cell line blood-brain barrier models. TEER after subtraction of blank transwells® of triplicate 24-transwells® and quadruplicate 96-transwells® was measured and displayed as averages from day 2 until day 7 after seeding and full assembly of cell layers. Error bars indicate the respective standard deviation. hCMEC/D3 did not display a TEER above 50 Ω cm^2^. TEER was used as an indicator for the optimal day of pharmaceutical permeability testing for *in vivo-in vitro* validation.

**Fig. 3:**
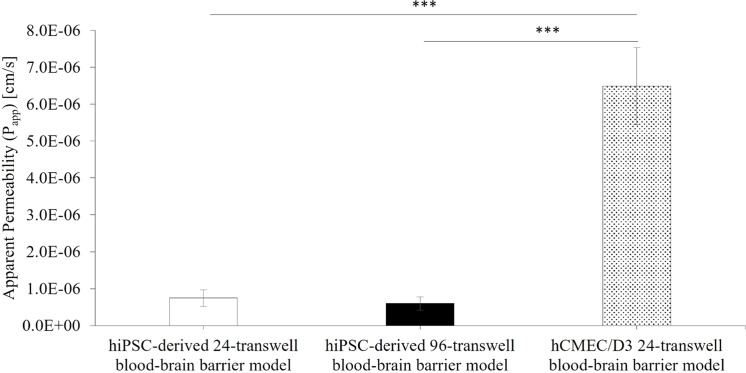
Paracellular marker permeability of hiPSC-derived and cell line blood-brain barrier (BBB) models. For hiPSC-derived models, diffusion of sodium fluorescein (10 µM) from donor to acceptor compartment was analyzed after 60 min incubation with a fluorescence reader at 485/20 nm excitation and 530/20 nm emission. The hCMEC/D3 cell line model was investigated by executing the same assay with lucifer yellow as a paracellular permeability marker (485/20 nm excitation and 527/20 nm emission). Bars represent the average apparent permeability ($P_{\text{app}}$) and standard deviation of triplicate 24-transwells® hiPSC-derived models, quadruplicate 24-transwell® cell line models and 6 biological replicates for the 96-transwell® hiPSC model. Statistical significance was calculated using the independent samples T-test (***p < 0.001).

The restrictive capacity of *in vitro* BBB models was investigated in terms of TEER and paracellular marker permeability. Monitoring TEER from day 2 until day 7 after seeding revealed that only tricellular hiPSC-derived BBB models displayed an *in vivo*-like TEER of at least 1 000 Ω cm^2^ (Fig. 2). The cell line model did not reach a TEER of above 50 Ω cm^2^ at any time during the recording. As opposed to the hiPSC-derived 24-transwell® model, the hiPSC-derived 96-transwell® model did show *in vivo*-like TEER immediately on day 2 until day 5 and a decline as of day 6. The 24-transwell® model achieved the *in vivo* benchmark on day 4 and remained close to 2 500 Ω cm^2^ until the end of the measurement period. Paracellular marker permeability analysis showed similar patterns of barrier integrity including $P_{\text{app}}$ below 1 × 10^–6^ cm/s for both hiPSC-derived models and above 6 × 10^–6^ cm/s for the cell line model (Fig. 3). In addition, unpublished immunofluorescence data (Supplementary Data Fig. 2) using FCDI hiPSC-derived BMECs, astrocytes and pericytes showed that the cells express cellular marker proteins as disclosed by the respective certificates of analysis (Supplementary Data File 1). This included P-gp and TFRC for BMECs, GFAP (glial fibrillary acidic protein, astrocyte marker protein) for astrocytes and NG2 (neural/glial antigen 2, pericyte marker protein) for pericytes.

### 3.2 Permeability testing and *in vivo-in vitro* validation

**Fig. 4:**
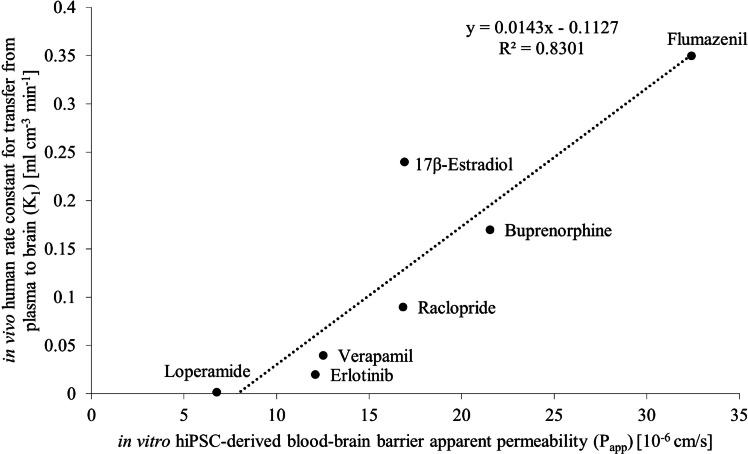
*In vivo-in vitro* validation of the tricellular hiPSC-derived blood-brain barrier (BBB) model with human clinical data. Apparent permeability ($P_{\text{app}}$) of pharmaceutical compounds (5 µM Loperamide, 5 µM Verapamil, 5 µM Erlotinib, 5 µM Raclopride, 5 µM Flumazenil, 10 µM 17β-Estradiol and 5 µM Buprenorphine) was investigated by application in the basolateral compartment and sampling after 60 min from both sides of 96-transwells®. The compound concentration was analyzed by liquid chromatography-high resolution mass spectrometry (LC—HRMS/MS). $P_{\text{app}}$ was plotted as the average from quadruplicate 96-transwells® against human *in vivo* rate constant for compound transfer from arterial blood across the BBB into brain ($K_{1}$) [mL cm^-3^ min^-1^]. A linear model was fitted along with the respective coefficient of determination (R^2^). The permeability of the radioactively labelled pesticide [benzyl-^14^C]-Deltamethrin (4417 Bq/mL (3 µM)) was examined with the same 60 min-assay and analyzed by scintillation counting. The human *in vivo*$K_{1}$ was predicted utilizing the estimates of the linear model.

The examination of the *in vitro* apparent permeability of pharmaceuticals revealed limited penetration of the hiPSC-derived BBB by Loperamide, Erlotinib, and Verapamil with $P_{\text{app}}$ < 15 × 10^–6^ cm/s (Fig. 4 and Supplementary Data Table 2). Elevated permeability of $P_{\text{app}}$ > 15 × 10^–6^ cm/s was measured for Raclopride, 17β-Estradiol and Buprenorphine. Flumazenil displayed the highest permeability of all tested compounds ($P_{\text{app}}$ > 30 × 10^–6^ cm/s). Plotting *in vitro* $P_{\text{app}}$ against published human *in vivo* BBB permeability $K_{1}$ [mL cm^-3^ min^-1^] data (Ghazanfari et al., 2024; Le Roux et al., 2019) showed a highly significant correlation in terms of the Spearman’s rank correlation coefficient of 0.964** (p < 0.01) (Fig. 4). In addition, the coefficient of determination (R^2^ = 0.8301) of the respective linear model indicated that a large proportion of the variance of the *in vivo* $K_{1}$ can be explained by the variance of *in vitro* $P_{\text{app}}$. This meant that within the linear model presented here, *in vitro* $P_{\text{app}}$ could be a successful predictor of *in vivo* $K_{1}$. The permeability case study of [benzyl-^14^C]-Deltamethrin revealed a very low penetration through the *in vitro* BBB ($P_{\text{app}}$ = 0.979 × 10^–6^ cm/s) (Fig. 4 and Supplementary Data Table 2). After insertion of $P_{\text{app}}$ into the linear model, *in vivo* $K_{1}$ was predicted to be close to 0 (−0.099 mL cm^-3^ min^-1^). LSC revealed that more than 75 % radioactivity of the [benzyl-^14^C]-Deltamethrin application stock was detected and thus, bioavailable within basolateral and apical media volumes (Supplementary Data File 3).

**Fig. 5:**
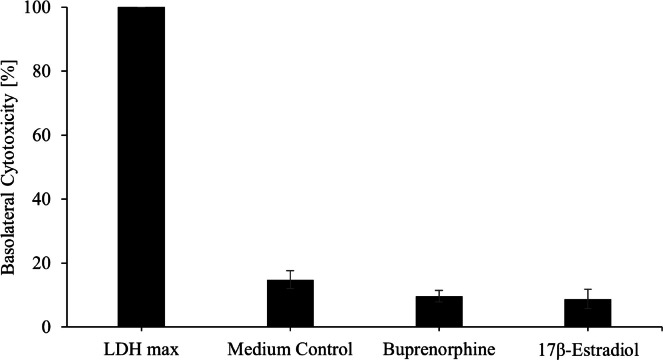
Cytotoxicity of representative high-permeability compounds in permeability studies. For maximum lactate dehydrogenase (LDH max) release 50 % of apical and basolateral volumes were replaced with lysis buffer. After incubation for 45 min at 37 °C and 5 % CO_2_, absorbance was measured at 450 nm and corrected for background signal (690 nm) and blank measurements. Bars represent mean cytotoxicity (and standard deviation) of six biological replicates as the percentage of maximum LDH release in the BMEC compartment.

The investigation of cytotoxicity did not result in the detection of elevated LDH-release after compound incubation for permeability testing (Fig. 5). Furthermore, base level cytotoxicity in medium controls was clearly below 20 % relative to lysed cells.

## 4 Discussion

The first goal of this study was to redesign a commercially available human *in vitro* BBB model and permeability testing method using characterized and published human cells for animal-free brain penetration assessment in regulatory neurotoxicity studies. Consequently, after redesigning, the BBB model should allow for standardization, be readily accessible to other laboratories, provide sufficiently high throughput, and suit industrial (regulatory) schedules. An initial comparison of the hCMEC/D3 cell line and a hiPSC-derived BBB model revealed that only the stem cell-derived model displayed *in vivo*-like barrier integrity (TEER > 1 000 Ω cm^2^, fluorescent marker permeability of 0.5 × 10^–6^ cm/s to 2 × 10^–6^ cm/s) as required in the regulatory context (Abbott et al., 2022; Escher et al., 2022). The final model presented here emulated complex human physiology by the inclusion of three hiPSC-derived BBB cell types in a scalable 96-transwell® format. To our knowledge, this is the first human *in vitro* BBB model for brain penetration assessment that achieved *in vivo*-like barrier properties using cryopreserved hiPSC-derived cells within 5 days and could therefore be assayed within one industrial working week. Importantly, the redesigned model including BMECs on the basolateral side also allows for direct transfer into micro physiological systems (MPS) such as the TissUse HUMIMIC chip and thus, exposure to shear stress, which is an important *in vivo*-like feature for future investigations.

Previously, the culture of hiPSC-derived models has been connected to a labor-intensive, multi-week protocol due to cell differentiation and expansion schedules, often reduced cellular complexity, and a lack of potential to cryopreserve differentiated hiPSC-derived BBB cells (Appelt‐Menzel et al., 2018; Le Roux et al., 2019; Stebbins et al., 2016). In this study, recent advances in cryopreservation protocols enabled a drastic reduction of accessibility hurdles for hiPSC-derived BBB cells, workload, and set-up time (Fig. 1) (Jezdić et al., 2025; Stanković et al., 2025). In addition, the utilization of cryopreserved cells allowed for increased cellular complexity in terms of three BBB cell types, and thus, added physiological relevance (Fig. 1) (Mármol et al., 2023). Consequently, the redesigned model and 5-day protocol presented here demonstrated an advanced application of hiPSC-technology leading to improved biomimetic potential, which has been required for acceptance of NAMs in regulatory neurotoxicity hazard assessment (Escher et al., 2022; Fritsche and Hogberg, 2020).

Given that fully differentiated hiPSC-derived BBB cells are commercially available, different laboratories can utilize the same resources, which should foster the standardization and validation of NAMs. This has been highlighted as another critical factor for regulatory acceptance of BBB *in vitro* models and for the replacement of animal testing (Abbott et al., 2022; Crofton et al., 2022; Escher et al., 2022). Nevertheless, a larger market of male and female cryopreserved resources would be desirable to foster generalizability, account for donor effects, reduce costs and promote throughput, which currently still limits the utilization of cryopreserved, hiPSC-derived cells for brain penetration assessment (Seo et al., 2020). In addition, current hiPSC differentiation protocols still lead to hiPSC-derived BMECs expressing epithelial proteins, which points to the model like- character of hiPSC-derived BBB models (Abbott et al., 2022; Delsing et al., 2020). As a result, consistent future success of recent methodological efforts to overcome respective non-BBB features would be helpful to foster model acceptance (Abbott et al., 2022; Delsing et al., 2020).

The *in vitro* barrier integrity of thawed hiPSC-derived BBB cells closely resembled leak-tight properties of advanced non-freezing models with peak TEER at day 2 or 3 after seeding of BMECs (Fig. 2) (Fengler et al., 2022; Stebbins et al., 2016). Similarly, extremely low fluorescent marker permeability confirmed peak TEER as an indicator of tight maturation of the *in vitro* barrier (Fig. 3), which has been proposed as an important control before permeability testing (Stebbins et al., 2016). As opposed to previous models, it became possible to raise the barrier quality control benchmark for individual models to be included in permeability experiments to *in vivo standards* (TEER > 1 000 Ω cm^2^, fluorescent marker permeability of 0.5 × 10^–6^ cm/s to 2 × 10^–6^ cm/s) (Koenig et al., 2022; Le Roux et al., 2019). Respective preconditions strongly limit paracellular permeation and the potential for artifacts in permeability tests, which fosters human relevance of the *in vitro* model (Abbott et al., 2022).

The second goal of this study was to validate the hiPSC-derived BBB model with human clinical PET data to enable reliable prediction of *in vivo* BBB permeability in regulatory neurotoxicity hazard assessment. Like previous models, the correlation between *in vivo* and *in vitro* data and the R^2^ of the derived linear model were indicative of *in vitro* $P_{\text{app}}$ being well-suited to predict *in vivo* permeability $K_{1}$ (Fig. 4) (Le Roux et al., 2019; Mabondzo et al., 2010). This is supported by the lowest predicted permeability for the known efflux transporter substrates Loperamide, Verapamil, and Erlotinib, which have been clinically shown to enter the human brain relatively poorly ($K_{1}$ < 0.05 mL cm^-3^ min^-1^) (Bauer et al., 2015; Traxl et al., 2015). Even though, these results recapitulate the effects of *in-vivo* like active efflux, additional future investigations of polarized efflux transporter activity would foster additional characterization of the utilized cellular model (Le Roux et al., 2019). Compared to previous hiPSC-derived BBB results, this study did not find additional evidence that Erlotinib displays high *in vitro* permeability ($P_{\text{app}}$ > 15 × 10^–6^ cm/s), which has been linked to potential efflux transporter inhibition before (Le Roux et al., 2019). At the other extreme, our *in vitro* model confirmed elevated human *in vivo* permeability ($K_{1}$ > 0.1 mL cm^-3^ min^-1^) of 17β-Estradiol, Buprenorphine and Flumazenil (Delforge et al., 1995; Le Roux et al., 2019; Zubieta et al., 2000). In this context, the case of 17β-Estradiol is particularly notable, as this study provides new human-relevant evidence that estrogens can penetrate the brain at a relatively high rate, which is a key prerequisite for their function as neuroactive steroids (Ghazanfari et al., 2024; Melcangi, 2024). Importantly, the investigation of cytotoxicity of high-permeability compounds showed that brain penetration was not related to cytotoxic effects (Fig. 5). Together, these findings provide support that the redesigned model presented here appears suitable to distinguish CNS from non-CNS compounds and that it recapitulates the effects of efflux transport for well-known substrates (Bauer et al., 2015; Le Roux et al., 2019; Mabondzo et al., 2010; Traxl et al., 2015). This conclusion is limited by the scarcity of clinical PET study data for IVIV-comparison to further increase reliable prediction of the *in vivo* state. Consequently, we support the notion from previous researchers that more PET investigations of human brain penetration would be desirable in the future (Abbott et al., 2022).

In the absence of human data, the low passage of the highly lipophilic insecticide Deltamethrin (log *P* = 6.1) through the *in vitro* BBB was in line with previous examinations in rats, where Deltamethrin entered the brain and exhibited neurotoxic effects only after 10 mg/kg oral dosing (Amaraneni et al., 2016; Kim et al., 2008; Kim et al., 2007). These findings add to former results, which found a parabolic relationship between lipophilicity and brain penetration, with limited BBB passage for highly lipophilic compounds (Amaraneni et al., 2016; Rowley et al., 1997). Conclusively, no additional support was found for Deltamethrin to be not only neurotoxic for insects but also brain penetrating in humans (Verma, 2024). In the future, more case studies are needed to investigate the properties of potentially brain-penetrating compound groups to foster the relevance of our model for different chemical structures. In this context, the enormous number of animal-based registration studies previously performed by chemical companies can provide a valuable source for cross-comparisons if human data is not available. For direct investigation of neurotoxic effects in humans, future inclusion of microglia, oligodendrocytes or neurons into our model could add relevant dimensions for regulatory neurotoxicity studies (Pérez-López et al., 2023). Depending on the study objectives, not only neurotoxicity readouts but also specific neurotoxic mechanisms such as neuroinflammation could be examined (Pérez-López et al., 2023).

## 5 Conclusion

In summary, the redesigned human *in vitro* BBB model presented here emulates key structural and functional characteristics of complex human *in vivo* BBB physiology. The utilization of the novel potential of commercially available, cryopreserved hiPSC-derived BBB cells and *in vivo* human PET data for permeability validation can enable reliable prediction of human *in vivo* brain penetration. Overall, our results suggest that the collective BBB model and brain penetration testing method could be an important starting point for a standardizable NAMs application to be implemented in regulatory neurotoxicity hazard assessment.

For future research, additional characterization of the tricellular model including polarized efflux transporter activity would further increase the physiological relevance of the BBB model. Besides, continuous validation with new clinical PET data would be desirable to foster model reliability. Furthermore, the redesigned model enables direct transfer into an MPS such as the TissUse HUMIMIC chip to include exposure to shear stress. This could be an important advancement in terms of *in vivo* modeling. Finally, the integration of the presented *in vitro* methods with future *in silico* techniques could enhance the creation of more reliable predictive models, potentially decreasing the reliance on animal testing. As we deepen our understanding of BBB mechanisms through these innovative strategies, we may pave the way for more efficient processes in drug development and regulatory safety evaluations.

## Funding

This work was financially supported by the Bayer AG Division Crop Science, Monheim, Germany.

## CRediT authorship contribution statement

**Paul Kurtenbach:** Writing – original draft, Visualization, Validation, Project administration, Methodology, Investigation, Formal analysis, Data curation, Conceptualization. **Sam Thilmany:** Validation, Formal analysis. **Maria Hahn:** Writing – review & editing, Supervision, Methodology. **Heidrun Ellinger-Ziegelbauer:** Investigation, Resources, Validation. **Andreas Thomas:** Writing – review & editing, Methodology. **Marc Lamshöft:** Writing – review & editing, Supervision, Project administration, Methodology, Funding acquisition. **Mario Thevis:** Writing – review & editing, Supervision, Resources, Funding acquisition.

## Declaration of competing interest

The authors declare the following financial interests/personal relationships which may be considered as potential competing interests:

Paul Kurtenbach reports financial support was provided by Bayer AG Division Crop Science, Monheim, Germany. Paul Kurtenbach reports a relationship with Bayer AG Division Crop Science, Monheim, Germany that includes: employment. Maria Hahn reports a relationship with Bayer AG Division Crop Science, Monheim, Germany that includes: employment. Marc Lamshoeft reports a relationship with Bayer AG Division Crop Science, Monheim, Germany that includes: employment. Heidrun Ellinger-Ziegelbauer reports a relationship with Bayer AG Pharmaceuticals, Wuppertal, Germany that includes: employment. If there are other authors, they declare that they have no known competing financial interests or personal relationships that could have appeared to influence the work reported in this paper.

## Data Availability

Data is contained within the article or supplementary material.

## Appendix Supplementary materials

Image, application 1

Image, image 2

Image, application 3

Image, image 4

Image, application 5

Image, application 6

Image, application 7

Image, application 8

Image, application 9

Image, application 10

Image, application 11
