# Supplementary material for: A cryopreserved and in vivo-in vitro validated human induced pluripotent stem cell blood-brain barrier model for reliable neurotoxicity assessment
Source: NAM J. 2025 Jul 17;1:100039. doi: 10.1016/j.namjnl.2025.100039 (PMC13288645; doi:10.1016/j.namjnl.2025.100039)
Supplement: Supplementary file 1 [file mmc1.docx]

**Supplementary Data Figure 1. Microscopy images of the 96‑transwell^®^ hiPSC‑derived blood-brain barrier model after full assembly**

Photos were taken after seeding of all cells of the 96‑transwell^®^ hiPSC‑derived BBB model (day 0) using a Zeiss Primovert microsope to inspect the cell layers for obvious leakages or detachment of cells. (A) Blank transwell^®^ filter. (B) Tricellular hiPSC‑derived blood-brain barrier model.
