# Supplementary figures and images for: A cryopreserved and in vivo-in vitro validated human induced pluripotent stem cell blood-brain barrier model for reliable neurotoxicity assessment
Source: NAM J. 2025 Jul 17;1:100039. doi: 10.1016/j.namjnl.2025.100039 (PMC13288645; doi:10.1016/j.namjnl.2025.100039)

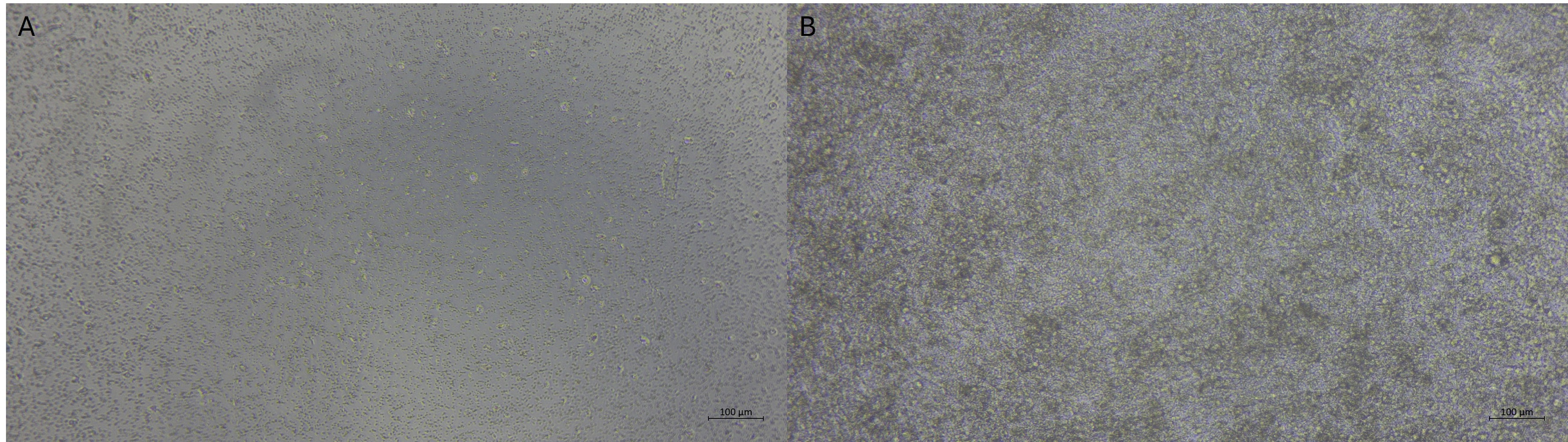

Supplement: Supplementary file 2 [file mmc2.jpg]

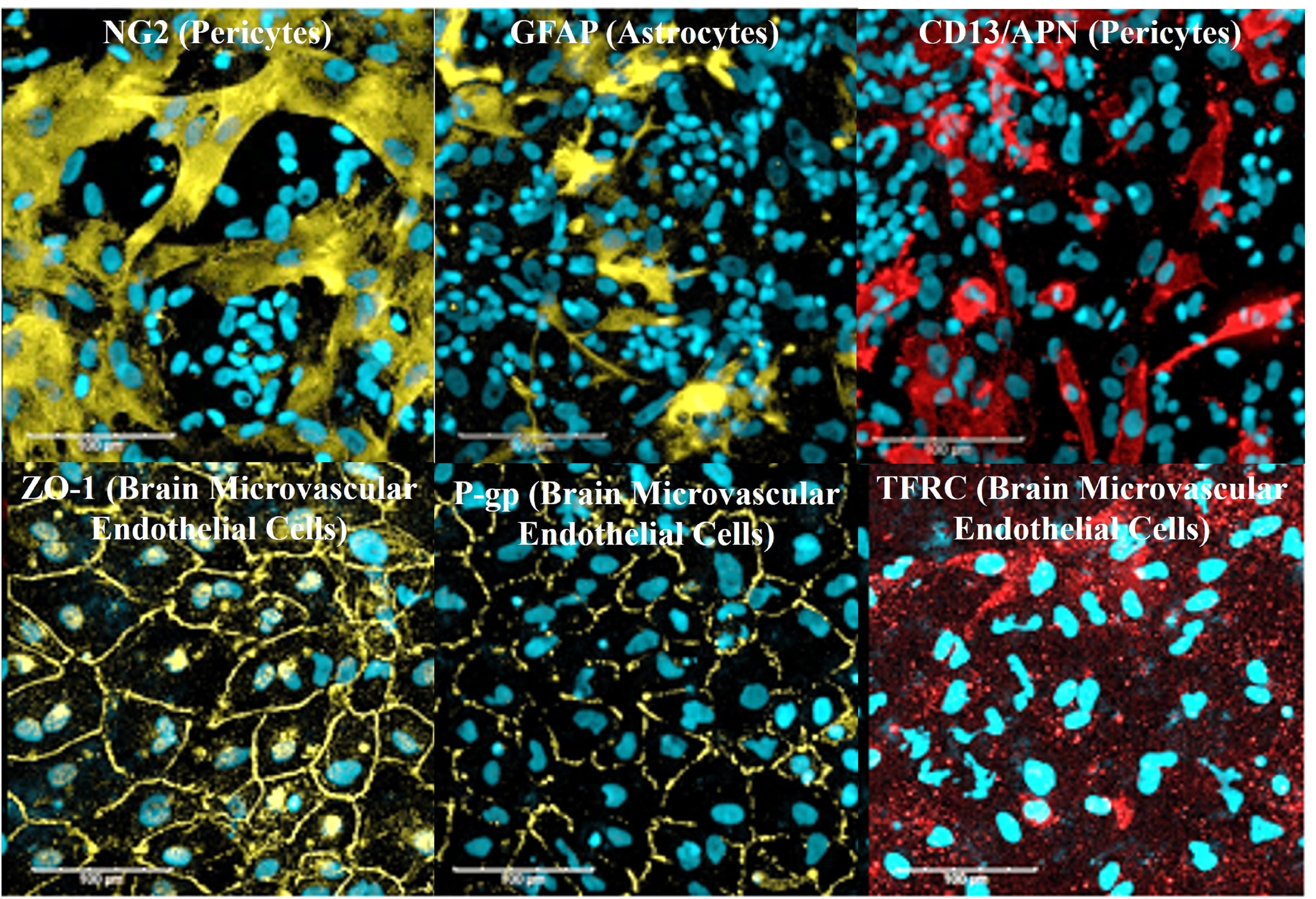

Supplement: Supplementary file 4 [file mmc4.jpg]
