# Supplementary material for: A cryopreserved and in vivo-in vitro validated human induced pluripotent stem cell blood-brain barrier model for reliable neurotoxicity assessment
Source: NAM J. 2025 Jul 17;1:100039. doi: 10.1016/j.namjnl.2025.100039 (PMC13288645; doi:10.1016/j.namjnl.2025.100039)
Supplement: Supplementary file 3 [file mmc3.docx]

**Supplementary Data Figure 2. Unpublished immunofluorescence data of hiPSC‑derived blood-brain barrier (BBB) cells**

Immunofluorescence stainings show expression of the marker proteins NG2 (neural/glial antigen 2, pericyte marker protein), GFAP (glial fibrillary acidic protein, astrocyte marker protein), CD13/APN (Aminopeptidase N, pericyte marker protein), ZO-1 (zonula occludens-1, tight-junction marker protein for brain microvascular endothelial cells (BMECs)), P-gp (permeability glycoprotein 1, efflux transporter marker protein for BMECs) and TFRC (transferrin receptor protein 1, receptor-mediated transcytosis marker protein for BMECs) in the respective cell types.

The scale bar equals 100 µm. The methodological background of the data is briefly summarized as follows: hiPSC‑derived brain microvascular endothelial cells (BMECs), astrocytes and pericytes were acquired from Fujifilm Cellular Dynamics Inc. (FCDI, Madison, WI, US, catalogue: R1241) as part of a BBB kit. hiPSC‑derived microglia and glutaneurons were acquired individually from FCDI. BMECs were seeded into the bottom channel of a previously published microphysiological system (MPS) by Emulate Inc. (Boston, MA, USA) (Vatine et al. 2019). On the following day, astrocytes, pericytes, glutaneurons and microglia were seeded into the top channel. 6 days after co‑coculture and 5 days after initiation of exposure to flow, the immunofluorescence analysis was performed according the instructions by Emulate (2025) using the following primary antibodies: anti‑GFAP (Abcam catalogue: ab68428, 1:100), anti‑NG2 (Abcam catalogue: ab275024, 1:50), anti‑CD13 (Abcam catalogue: ab7417, 1:1000), anti‑ZO1 (Thermo Fisher Scientific catalogue: 61‑7300; 1:100), anti P‑gp (Abcam catalogue: ab170904; 1:500), anti‑TFRC (Abcam catalogue: ab9179, 1:1000). As secondary antibodies Alexa Fluor® 647 (red staining) and Alexa Fluor® 549 (yellow staining) were used.

**References**

Emulate, 2025. Protocol for Emulate Organ-Chips: Fixation and Immunofluorescence (IF) Staining. https://emulatebio.com/wp-content/uploads/2021/06/EP137_v1.0_Fixation_and_Immunofluorescence-IF-Staining.pdf (accessed 30.06.2025, 2025).

Vatine, G.D., Barrile, R., Workman, M.J., Sances, S., Barriga, B.K., Rahnama, M., Barthakur, S., Kasendra, M., Lucchesi, C. and Kerns, J. 2019. Human iPSC-derived blood-brain barrier chips enable disease modeling and personalized medicine applications. Cell stem cell 24, 995-1005. e1006, doi: 10.1016/j.stem.2019.05.011.
