# Supplementary material for: A cryopreserved and in vivo-in vitro validated human induced pluripotent stem cell blood-brain barrier model for reliable neurotoxicity assessment
Source: NAM J. 2025 Jul 17;1:100039. doi: 10.1016/j.namjnl.2025.100039 (PMC13288645; doi:10.1016/j.namjnl.2025.100039)
Supplement: Supplementary file 5 [file mmc5.docx]

**Supplementary Data File 1. Certificates of analysis for hiPSC-derived brain microvascular endothelial cells (BMECs), pericytes and astrocytes**

The certificates of analysis (CoA) show that the cellular identity of all three cell types of the tricellular blood-brain barrier (BBB) model is supported by the expression cell-specific markers, which were analyzed by flow cytometry. The CoA for BMECs displays that the cells express multiple key transporter proteins including permeability glycoprotein 1 (PGP, MDR1), glucose transporter 1 (GLUT1), CD98 (SLC3A2) and transferrin receptor protein 1 (TFRC/TfR1), which represent all major BMEC transport mechanisms in terms of active efflux (PGP), carrier-mediated transport (GLUT1 and CD98) and receptor-mediated transcytosis (TFRC). Pericytes were identified by the expression of two brain pericyte markers in terms of platelet-derived growth factor receptor beta (PDGFRβ) and neural/glial antigen 2 (NG2). Astrocytes were shown to express the astrocyte-specific marker glial fibrillary acidic protein (GFAP).
