# Supplementary material for: A cryopreserved and in vivo-in vitro validated human induced pluripotent stem cell blood-brain barrier model for reliable neurotoxicity assessment
Source: NAM J. 2025 Jul 17;1:100039. doi: 10.1016/j.namjnl.2025.100039 (PMC13288645; doi:10.1016/j.namjnl.2025.100039)
Supplement: Supplementary file 6 [file mmc6.pdf]

FUJIFILM
Cellular Dynamics

FUJIFILM Cellular Dynamics, Inc.
525 Science Drive
Madison, WI 53711 USA

Toll Free in US (877) 320-6688 / (608) 310-5100 **T**
(608) 310-5101 **F**
fcdi-support@fujifilm.com **E**
www.fujifilmcdi.com **W**

Certificate of Analysis

**Product Name:**	iCell® BMEC	**Lot Number:**	106138
**Catalog Number:**	C1239	**Unit Size:**	≥ 3 x 10^6 cells/vial
**Donor:**	01279	**Use By:**	12 months from date of shipment
**Phenotype:**	AHN		
		**Storage Conditions:**	Vapor Phase LN_2

This product is subject to use restrictions, which can be found in FUJIFILM Cellular Dynamics, Inc. Standard Terms and Conditions accessible at https://fujifilmcdi.com/assets/tnc/standard.pdf. Failure to adhere to these use restrictions, among other things, will void the limited warranty applicable to this product.

For further assistance, contact technical support (fcdi-support@fujifilm.com).

Test Parameter	Result
**Viable Cells**	3.8x10^6 cells at thaw by trypan blue exclusion
**Purity**	5 days post-thaw by flow cytometry
	95% GLUT1
	91% TFRC
	93% CD98
	83% MRP1
	94% PGP
**Viability**	69% at thaw by trypan blue exclusion
**Identity Confirmation**	Match by SNP genotyping
**Mycoplasma**	Pass by PCR Assay
**Sterility**	Pass per 21 CFR 610.12
**Barrier Function/Transepithelial Electrical Resistance (TEER)**	>1514 Ohms.cm^2

iPSC Line Authentication Statement

All FUJIFILM Cellular Dynamics, Inc. (FCDI) products are manufactured from tissue-derived induced pluripotent stem cell (iPSC) lines that were produced by FCDI. Each tissue sample received by FCDI (an Original Sample) is assigned a unique 5-digit identification code (ID). FCDI uses SNP analysis on genomic DNA (gDNA) isolated from the Original Sample to establish a DNA fingerprint for each ID.

The iPSC line and iPSC-derived differentiated cell type produced are identified by the Original Sample's ID and DNA fingerprinted to confirm identity with that Original Sample.

In the case a gene-edited iPSC is produced from a particular Original Sample, it is identified by the Original Sample's ID and confirmed using the DNA fingerprint. The gene-editing of the iPSC is confirmed using sequence analysis of the gene-edited region.

This lot passes all quality control specifications.

Quality Assurance Approval: [signature] Date: 12 Jan 2023

For Research Use Only. Not for use as a therapeutic or diagnostic.

© 2019 FUJIFILM Cellular Dynamics, Inc.

FUJIFILM
Cellular Dynamics

FUJIFILM Cellular Dynamics, Inc.
525 Science Drive
Madison, WI 53711 USA

Toll Free in US (877) 320-6688 / (608) 310-5100 **T**
(608) 310-5101 **F**
fcdi-support@fujifilm.com **E**
www.fujifilmcdi.com **W**

Certificate of Analysis

**Product Name:**	iCell® Pericytes	**Lot Number:**	106379
**Catalog Number:**	C1241	**Unit Size**	≥1.5x10^6 cells/vial
**Donor ID:**	01279	**Use By:**	12 months from date of shipment
**Phenotype:**	AHN	**Storage Conditions:**	Vapor Phase LN_2

This product is subject to use restrictions which can be found in FUJIFILM Cellular Dynamics, Inc. Standard Terms and Conditions accessible at https://fujifilmcdi.com/assets/tnc/standard.pdf. Failure to adhere to these use restrictions, among other things, will void the limited warranty applicable to this product.

For further assistance, contact technical support (fcdi-support@fujifilm.com).

Test Parameter	Result
**Viable Cells**	1.7x10^6 cells at thaw by Trypan blue exclusion
**Purity,**	5 days post-thaw by Flow Cytometry
	93% PDGFR-β (CD140b)
	87% NG2
	84% CD13
	99% Desmin
	98% αSMA
**Viability**	70% at thaw by Trypan blue exclusion
**Identity Confirmation**	Match by SNP genotyping
**Mycoplasma**	Pass by PCR Assay
**Sterility**	Pass per 21 CFR 610.12

iPSC Line Authentication Statement

All FUJIFILM Cellular Dynamics, Inc. (FCDI) products are manufactured from tissue-derived induced pluripotent stem cell (iPSC) lines that were produced by FCDI. Each tissue sample received by FCDI (an Original Sample) is assigned a unique 5-digit identification code (ID). FCDI uses SNP analysis on genomic DNA (gDNA) isolated from the Original Sample to establish a DNA fingerprint for each ID.

The iPSC line and iPSC-derived differentiated cell type produced are identified by the Original Sample's ID and DNA fingerprinted to confirm identity with that Original Sample.

In the case a gene-edited iPSC is produced from a particular Original Sample, it is identified by the Original Sample's ID and confirmed using the DNA fingerprint. The gene-editing of the iPSC is confirmed using sequence analysis of the gene-edited region.

This lot passes all quality control specifications.

Quality Assurance Approval: [illegible] Date: 04Jan2023

For Research Use Only. Not for use as a therapeutic or diagnostic. TEMP-00034.01 Effective Date:30 Sep 2019
© 2019 FUJIFILM Cellular Dynamics, Inc.
Rev 00

FUJIFILM
Cellular Dynamics

FUJIFILM Cellular Dynamics, Inc.
525 Science Drive
Madison, WI 53711 USA

Toll Free in US (877) 320-6688 / (608) 310-5100 **T**
(608) 310-5101 **F**
fcdi-support@fujifilm.com **E**
www.fujifilmcdi.com **W**

Certificate of Analysis

**Product Name:**	iCell® Astrocytes 2.0	**Lot Number:**	106977
**Catalog Number:**	C1249	**Unit Size**	≥1x10^6 cells/vial
**Donor ID:**	01279	**Use By:**	12 months from date of shipment
		**Storage Conditions:**	Vapor Phase LN_2

This product is subject to use restrictions which can be found in FUJIFILM Cellular Dynamics, Inc. Standard Terms and Conditions accessible at https://fujifilmcdi.com/assets/tnc/standard.pdf. Failure to adhere to these use restrictions, among other things, will void the limited warranty applicable to this product.

For further assistance, contact technical support (fcdi-support@fujifilm.com).

Test Parameter	Result
**Viable Cells**	1.6x10^6 cells by trypan blue exclusion
**Purity, 7 days post thaw**	>99% CD44$^+$
	97% CD49f$^+$
	75% GFAP$^+$
	8% DCX$^+$
**Viability**	86% at thaw, by trypan blue exclusion
**Identity Confirmation**	Match by SNP genotyping
**Mycoplasma**	Pass by PCR
**Sterility**	Pass following USP71 Modified
**Glutamate Uptake**	Pass 7 days post-thaw

iPSC Line Authentication Statement

All FUJIFILM Cellular Dynamics, Inc. (FCDI) products are manufactured from tissue-derived induced pluripotent stem cell (iPSC) lines that were produced by FCDI. Each tissue sample received by FCDI (an Original Sample) is assigned a unique 5-digit identification code (ID). FCDI uses SNP analysis on genomic DNA (gDNA) isolated from the Original Sample to establish a DNA fingerprint for each ID.

The iPSC line and iPSC-derived differentiated cell type produced are identified by the Original Sample's ID and DNA fingerprinted to confirm identity with that Original Sample.

In the case a gene-edited iPSC is produced from a particular Original Sample, it is identified by the Original Sample's ID and confirmed using the DNA fingerprint. The gene-editing of the iPSC is confirmed using sequence analysis of the gene-edited region.

This lot passes all quality control specifications.

Quality Assurance Approval: [signature] Date: 31Aug2023

For Research Use Only. Not for use as a therapeutic or diagnostic. TEMP-00034.01 Effective Date:30 Sep 2019
© 2019 FUJIFILM Cellular Dynamics, Inc.
Rev 01
