# Supplementary material for: A cryopreserved and in vivo-in vitro validated human induced pluripotent stem cell blood-brain barrier model for reliable neurotoxicity assessment
Source: NAM J. 2025 Jul 17;1:100039. doi: 10.1016/j.namjnl.2025.100039 (PMC13288645; doi:10.1016/j.namjnl.2025.100039)
Supplement: Supplementary file 7 [file mmc7.docx]

**Supplementary Data File 2. Liquid chromatography‑high resolution mass spectrometry (LC‑HRMS/MS) method characterization**

Compounds were quantified utilizing standard calibration series, and quality control samples prepared in a blank matrix. An individual concentration range was established for each substance. Method characterization in terms of linearity, accuracy, and precision, was performed based on the analytical requirements issued by the German Society of Toxicological and Forensic Chemistry (GTFCh). Raw data was processed for quantification using the Quan Browser in Xcalibur (version 2.2) by Thermo Fisher Scientific. Peak areas were plotted against nominal concentrations to set up standard calibration curves, and linearity was confirmed by a coefficient of determination (R^2^) above 0.99. The respective linear functions were then used to calculate the analyte concentrations of unknown samples. For Buprenorphine and 17β‑Estradiol, the peak area ratios of analyte and internal standard were used analogously.
