# Supplementary material for: A cryopreserved and in vivo-in vitro validated human induced pluripotent stem cell blood-brain barrier model for reliable neurotoxicity assessment
Source: NAM J. 2025 Jul 17;1:100039. doi: 10.1016/j.namjnl.2025.100039 (PMC13288645; doi:10.1016/j.namjnl.2025.100039)
Supplement: Supplementary file 9 [file mmc9.docx]

**Supplementary Data File 3. Bioavailability of [benzyl-^14^C]-Deltamethrin**

Apparent permeability ($P_{\text{app}}$) of [benzyl-^14^C]-Deltamethrin was investigated by application in the basolateral compartment and sampling after 60 min from both sides of 96‑transwells^®^. The compound radioactivity of the application stock and samples was analyzed by liquid scintillation counting. The radioactivity of basolateral and apical samples was summed to calculate the total bioavailable radioactivity. Bars represent bioavailable [benzyl-^14^C]-Deltamethrin radioactivity and standard deviation of 4 replicates.
