# Supplementary material for: A cryopreserved and in vivo-in vitro validated human induced pluripotent stem cell blood-brain barrier model for reliable neurotoxicity assessment
Source: NAM J. 2025 Jul 17;1:100039. doi: 10.1016/j.namjnl.2025.100039 (PMC13288645; doi:10.1016/j.namjnl.2025.100039)
Supplement: Supplementary file 11 [file mmc11.docx]

**Supplementary Materials**

**A cryopreserved and *in vivo-in vitro* validated human induced pluripotent stem cell blood-brain barrier model for reliable neurotoxicity assessment**

Authors: Paul Kurtenbach^a,b^, Sam Thilmany^b,c^, Maria Hahn^a^, Heidrun Ellinger-Ziegelbauer^d^, Andreas Thomas^b^, Marc Lamshöft^a^ and Mario Thevis^b,e^

Corresponding author: Mario Thevis (thevis@dshs-koeln.de)

^a^Bayer AG Division Crop Science, Alfred-Nobel-Strasse 50, 40789 Monheim am Rhein, Germany

^b^Institute of Biochemistry/Center for Preventive Doping Research, German Sport University Cologne, Am Sportpark Müngersdorf 6, 50933 Cologne, Germany

^c^Federal Institute for Drugs and Medical Devices, Kurt-Georg-Kiesinger-Allee 3, 53175 Bonn, Germany

^d^Bayer AG Division Pharmaceuticals, 42096 Wuppertal, Germany

^e^European Monitoring Center for Emerging Doping Agents (EuMoCEDA), Cologne/Bonn, Germany

**Supplementary Data Table 1. Additional compound library data**

| **Compound** | **Molecular Weight [g mol^-1^]** | **LogP** | **Solvent used for initial dissolution of solid substances** | **Final solvent concentration in spiked medium [%]** |
| --- | --- | --- | --- | --- |
| Loperamide | 477 | 5.13 | DMSO | 0.3 |
| Verapamil | 454.6 | 2.15 | DMSO | 0.3 |
| Erlotinib | 393.4 | 2.7 | DMSO | 0.2 |
| Raclopride | 347.2 | 2.9 | DMSO | 0.2 |
| Flumazenil | 303.29 | 1.9 | DMSO | 0.2 |
| 17β-Estradiol | 272.4 | 4.01 | Methanol | 0.3 |
| Buprenorphine | 467.6 | 4.98 | Methanol | 0.2 |
| Deltamethrin | 505.2 | 6.1 | ACN | 0.3 |

The final solvent concentration in spiked medium [%] refers to the application solutions used for permeability assays.

**Supplementary Data Table 2. Permeability data for *in vivo‑in vitro* validation**

| **Compound** | ***In vitro* hiPSC-derived blood-brain barrier model apparent permeability  (P_app_) [10^-6^ cm/s]** | ***In vivo* human rate constant for compound transfer from blood across the BBB into brain  (K_1_) [mL min^-1^ cm^-3^]  from Ghazanfari et al. 2024, Le Roux et al. 2019** | **Prediction of  K_1_ [mL min^-1^ cm^-3^]** |
| --- | --- | --- | --- |
| Loperamide | 6.753 | 0.002 |  |
| Verapamil | 12.502 | 0.04 |  |
| Erlotinib | 12.084 | 0.02 |  |
| Raclopride | 16.818 | 0.09 |  |
| Flumazenil | 32.403 | 0.35 |  |
| 17β-Estradiol | 16.889 | 0.24 |  |
| Buprenorphine | 21.517 | 0.17 |  |
| Deltamethrin | 0.979 |  | -0.099 |

Apparent permeability ($P_{\text{app}}$) of pharmaceutical compounds (5 µM Loperamide, 5 µM Verapamil, 5 µM Erlotinib, 5 µM Raclopride, 5 µM Flumazenil, 10 µM 17β-Estradiol and 5 µM Buprenorphine) was investigated by application in the basolateral compartment and sampling after 60 min from both sides of 96‑transwells^®^. The compound concentration was analyzed by liquid chromatography-high resolution mass spectrometry (LC-HRMS/MS). $P_{\text{app}}$ was calculated as the average from quadruplicates. Human *in vivo* BBB permeability data of $K_{\text{1}}$ [mL cm^-3^ min^-1^] have been published previously (Ghazanfari et al. 2024; Le Roux et al. 2019). The permeability of the radioactively labelled pesticide [benzyl-^14^C]-Deltamethrin (4417 Bq/mL (3 µM)) was examined with the same 60 min-assay and analyzed by scintillation counting. The human *in vivo* $K_{\text{1}}$ was predicted utilizing the estimates of the linear model derived from plotting *in vitro* P_app_ against *in vivo* K_1_ of pharmaceutical compounds.

**References**

Ghazanfari, N., Doorduin, J., van der Weijden, C.W., Willemsen, A.T., Glaudemans, A.W., van Waarde, A., Dierckx, R.A. and de Vries, E.F. 2024. Pharmacokinetic Analysis of [18F] FES PET in the Human Brain and Pituitary Gland. Molecular Imaging and Biology 26, 351-359, doi: 10.1007/s11307-023-01880-z.

Le Roux, G., Jarray, R., Guyot, A.-C., Pavoni, S., Costa, N., Théodoro, F., Nassor, F., Pruvost, A., Tournier, N. and Kiyan, Y. 2019. Proof-of-concept study of drug brain permeability between in vivo human brain and an in vitro iPSCs-human blood-brain barrier model. Scientific Reports 9, doi: 10.1038/s41598-019-52213-6.
